# Mechanical Characteristics of Cement Paste in the Presence of Carbon Nanotubes and Silica Oxide Nanoparticles: An Experimental Study

**DOI:** 10.3390/ma14061347

**Published:** 2021-03-11

**Authors:** Moses Karakouzian, Visar Farhangi, Marzieh Ramezani Farani, Alireza Joshaghani, Mehdi Zadehmohamad, Mohammad Ahmadzadeh

**Affiliations:** 1 Department of Civil and Environmental Engineering and Construction, University of Nevada, Las Vegas, NV 89154, USA; 2 School of Chemical Engineering, College of Engineering, University of Tehran, Tehran 1417466191, Iran; 3 Zachry Department of Civil Engineering, Texas A&M University, College Station, TX 77843, USA; joshaghani@tamu.edu; 4 Department of Civil and Environmental Engineering, Louisiana State University, Baton Rouge, LA 70803, USA; mzadeh2@lsu.edu; 5 Department of Civil Engineering, Sharif University of Technology, International Campus, Tehran 9417-76655, Iran; m.ahmadzadeh@student.sharif.edu

**Keywords:** silica nanoparticles, carbon nanotube, compressive strength, flexural strength

## Abstract

Considering the remarkable characteristics of nanomaterials, previous research studies investigated the effects of incorporating different types of these materials on improving the concrete properties. However, further studies are required to evaluate the complementary hybridization and synergistic influence of nanomaterials. In this research, the combined effect of adding nano silica particles (NS) and multi-walled carbon nanotubes (MWCNT) on enhancing both the compressive and flexural strengths of the cement paste was investigated. Moreover, the morphology of the interface between cement paste and aggregates was studied by scanning electron microscopy (SEM). The mixtures were prepared using three different portions of MWCNT and NS. Electron microscopy images indicated a uniform distribution of nanoparticles in the cement matrix, enhanced hydration reactions, and increased density. Based on the experiments’ outcomes, the combined utilization of silica and carbon nanomaterials in the cement paste did not necessarily result in the maximum compressive and flexural strengths. Furthermore, it was observed that the use of higher percentages of pristine NS in the absence of MWCNT can lead to further enhancement of strength properties of the cement paste.

## 1. Introduction

Presently, nanotechnology has been gaining wide attention for the use in construction materials. The advent of this technology in the construction industry has also been accompanied by considerable advancements in the building industry. The ultimate goal of using nanomaterials in the construction industry is to develop high-performance building mixtures as multi-purpose materials [1,2]. Such multifunctional properties of nanomaterials are the most important advantages of using nanotechnology compared to other approaches and common materials [3,4,5,6]. Some shortcomings of Portland cement, such as low values of flexural strength, ductility, and toughness, along with the resistance issues in interaction with chemicals, raise concerns regarding the reliability and durability of this material [7,8,9,10,11,12,13,14]. Thus, using nanomaterials along with the cementitious materials can address these issues and significantly increase the mechanical properties of concrete.

In previous investigations, great attention has been paid to the effect of using nano silica particles (NS) on the mechanical properties of concrete and cement. In this regard, Li et al. [15] studied the effect of NS on the mechanical behavior and microstructure of ordinary Portland cement (OPC) binary blends. They reported that adding 1.0 wt.% of NS considerably reduces the setting time and improves OPC’s strength. Based on their observations using mercury intrusion porosimetry (MIP) test and scanning electron microscopy (SEM), NS also enhances the binding within the hydrates and decreases the porosity. In another effort, Sargam and Wang [16] investigated the compressive strength, heat of hydration, and flowability of cement paste in the presence of NS dispersed with seven dispersants. They used polycarboxylate ether (PCE) based superplasticizer, sodium dodecyl sulfate (SDS), and anionic surfactants, in addition to Tritons (TX114, TX100, and TX405) and Tweens (T20 and T40). Based on the outcomes of this study, the hydration between cement and NS can be accelerated using PCE along with another surfactant. Moreover, they asserted that the compressive strength could be improved by using higher space ratios of cement–TX405 paste. Najigivi et al. [17] conducted a study on the impacts of using two types of NS on the compressive strength and workability of binary blended concrete cured in lime and water solutions. They examined the use of SiO_2_ nanoparticles with different ratios. They reported a reduction in the workability of the binary blends using SiO_2_ nanoparticles. Following the outcome of the hardened concrete test, the optimal substitution level of the cement paste was reported equal to 1.0 wt.% of cement. The ultimate compressive strength of the blended concrete was measured at the replacement level of 2.0 wt.%. The forming of extra calcium-silicate-hydrate (C-S-H) had the main role in improving the mechanical characteristics of concrete using NS. Based on other research studies [18,19,20], the effects of NS on the physical properties of concrete are stronger than those of silica fume. Both physical and pozzolanic aspects of NS are critical in developing concrete with ultra-high-performance characteristics. Mohammadyan-Yasouj and Ghaderi [21] investigated the application of carbon nanotubes (CNT) along with basalt fiber (BF) and waste glass power (WGP) on concrete’s mechanical characteristics. They highlighted the ternary application of these materials in the presence of NS. According to the outcomes of flexural, compressive, and water absorption tests along with the results of microscopy images, they mentioned that, by adding 0.1% CNT, 0.2% BF, and 20% WGP, a denser concrete with higher mechanical properties could be produced. Stynoski et al. [22] performed a comprehensive study on OPC mortar containing CNT, silica fume, and carbon fibers in an effort to analyze the ductility and crack width of cement paste. They specifically considered the interfacial transition zone within hydrated cement and fibers that can include a higher amount of calcium hydroxide and affect the porosity. They reported that the use of silica fume improves the fracture performance of mixtures in the presence of carbon fibers and CNT. Wang et al. [23] stated that cement paste with nanoparticles had more densified structures compared to the plain cement paste due to the reduction in the amount of pores and the presence of higher amount of Ca(OH)_2_ crystals along with enhancing the C-S-H quantity.

The influence of incorporating dispersed CNT on the mechanical characteristics of the cement paste and concrete was further investigated in many studies. CNT’s diameter usually ranges 0.5–100 nm as a type of nanomaterials with single- or multi-cylindrical layers. CNTs are characterized by their mechanical properties with a tensile strength range close to 200 GPa, which is about 100 times higher than steel. Their elastic modulus is greater than the 1 TPa, although their density is almost 15% of steel. Application of nanotechnology and fibers to improve the mechanical properties of self-compacting concrete (SCC) were assessed in several studies [24,25,26,27,28]. Fly ash (FA) as a sustainable material [29] can also be used along with nanomaterials. Accordingly, Aydin et al. [30] investigated the synergistic effect of CNT and NS in the presence of FA on flexural and compressive strengths of SCC. They reported an adverse impact of using NS on fresh concrete’s properties, considering the high water requirement. Although, the combined effect of using FA and NS not only increased the SCC’s mechanical properties, but also reduced FA’s bleeding and segregation effects by 40%. Wille and Loh [31] examined the application of multi-walled carbon nanotubes (MWCNT) to enhance the bond within the steel fibers and the ultra-high-performance concrete’s (UHPC’s) matrix by improving the packing density. Following their study, a relatively low concentration of MWCNT (e.g., 0.022% wt.%) can considerably increase the bonding response of steel fibers. Wang et al. [32] asserted that the presence of CNT could improve OPC paste’s characteristics, such as flexural strength, modulus of elasticity, and electrical conductivity. Additionally, using CNT enhances sensitivity of the piezoresistive reaction to the propagation of microcracks in the cement paste. Such characteristics of CNT highlights its performance compared to other commonly used fibers to reinforce composites. The outstanding CNT properties strengthen the cement paste against the occurrence of early-age microcracks [33]. Joshaghani [34] assessed the performance of titanium dioxide (TiO_2_) and carbon-nanofibers (CNF) singularly and simultaneously. He reported that concrete samples containing CNF were improved in terms of mechanical properties. The reason was attributed to the bridging effect of the CNFs for microcracks and filler effect. Incorporating both TiO_2_ and CNF at the same time would not necessarily increase the strength. The decrease in the strength could be due to the agglomeration and deficiencies caused by the dispersion of TiO_2_ particles and CNF. Moreover, Kang et al. [35], and Vesmawala et al. [36] proposed a significant enhancement of cement mechanical properties by adding CNTs. In another research, Hu et al. and Vidivelli et al. [37,38] reported durability improvements using CNT in the cement composites. Konsta-Gdoutos et al. [39], and Praveen et al. [40] proposed significant porosity reduction and reinforced matrix enhancement by adding C-S-H gel. The application MWCNT resulted in further uniform distribution of pore size along with tension load-transfer by bridging action [41,42]. Additionally, CNT reduced the shrinkage in the heating procedure of the autoclaved aerated concrete (AAC) [43].

Developing hybrid materials using the nanotechnology method enhances both the performance and durability of the produced material [44,45]. It also provides an environmentally-friendly approach by minimizing natural resources as conventional construction materials [46,47,48,49,50]. Sikora et al. [51] investigated the effects of using MWCNT coated with NS on the cement pastes subjected to elevated temperature up to 600 °C. They found that incorporating MWCNT and NS with the optimum amount equal to 0.125 wt.% of the cement paste was advantageous compared to the use of pristine MWCNT. The research team found an increase in the binding ability within the nanotubes and cement matrices. On the other hand, excessive use of MWCNT and NS in the cement paste could reduce the thermal capacity due to the nanomaterial’s agglomeration. Narasimman et al. [52] studied the effects of incorporating combined CNT and NS materials on the lightweight concrete’s compressive strength. CNT and NS as nano reinforcement fillers were used along with expanded clay to improve the compressive strength by applying the ultrasonication method. They utilized different ratios of CNT and NS with the optimal replacement level of these reinforcement fillers equal to 3% of cement paste. The research team reported that the maximum 28-day compressive strength was 10.7 MPa by using 2% CNT and 1% NS. In addition, they asserted that using the pristine CNT adversely affected the 28-day compressive strength. Garg et al. [53] reported that the combination of NS with low fractions of CNT in the cement paste improved the durability of concrete and enhanced the sensing capability of the smart concrete.

These findings illustrate the remarkable advantages of utilizing different types of nanoparticles to enhance the concrete properties. However, more research studies are required to investigate the effects of using nanomaterials to address the potential deficiencies of cement paste and further strengthen the concrete characteristics. In the present study, the effects of different ratios of CNT and NS on the mechanical properties were investigated. In addition, the morphology was examined by transmission electron microscopy (TEM). Then, scanning electron microscope (SEM) images were analyzed to illustrate nanoparticles’ distribution in the cement matrix along with the filled pores. Finally, the compressive and flexural strength variations of the OPC paste in the presence of nanoparticles were tested.

## 2. Materials

Type II Portland cement type was used in this study, which was acquired from Abyek Company, Abyek, Iran. This type of cement was as a moderate sulfate resistant cement and moderate heat of hydration in accordance with American Society for Testing and Materials (ASTM) C150 specifications [54]. The properties of Portland cement are summarized in Table 1. Polycarboxylate-based superplasticizer was used along with water in the mixture. The required amount of water to perform the hydration reactions with the related compounds in the cement was taken into consideration. Following the ASTM C109 [55] standard, the water–cement and aggregate–cement ratios of 0.30 and 2.65 were used, respectively.

MWCNT were acquired from Sigma-Aldrich Company and NS were prepared by Shanghai Aladdin biochemical technology company, Shanghai, China. The physical properties of the MWCNT and NS are presented in Table 2.

**Figure 1:**
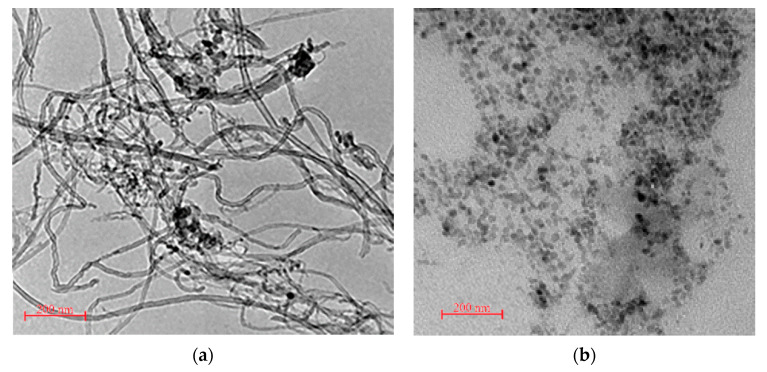
Morphology analysis with HRTEM: (**a**) CNT; and (**b**) nanoparticles SiO_2_ (NS).

The high-resolution transmission electron microscope (HRTEM) by the JEOL Ltd. (JEM-2100, Akishima, Japan) at 100 kV was utilized in this study to analyze the morphology of MWCNT. As shown in Figure 1a, MWCNT had a tubular shape with diameter ranging 20–40 nm. The HRTEM micrograph demonstrated that the obtained NS has a spherical shape with few agglomerations (see Figure 1b).

**Figure 2:**
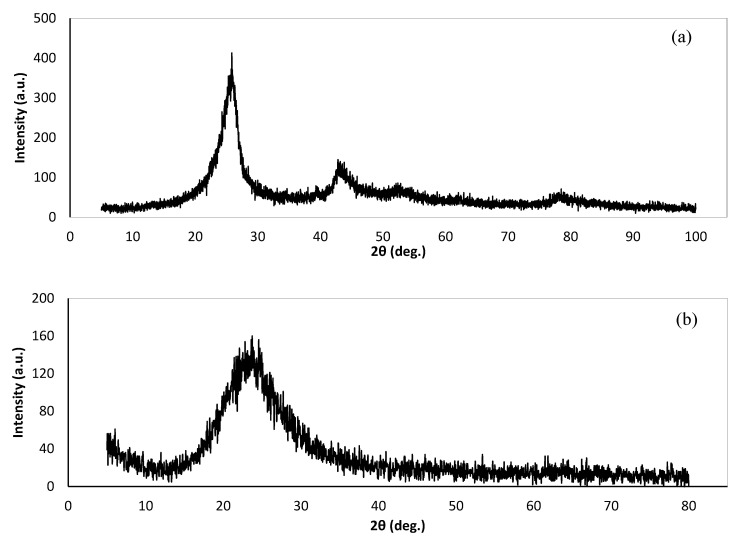
XRD diffraction pattern of: (**a**) CNT; and (**b**) SiO_2_ nanoparticles.

MWCNT were also examined by X-ray diffraction (XRD), to evaluate the mineral compositions using Bruker D8-Advance diffractometer with monochromatic CuKa radiation, Bruker Corporation, Billerica, Massachusetts, USA. The XRD patterns of MWCNT and silica oxide nanoparticles are presented in Figure 2a,b, respectively. The diffraction peaks at 26.2° and 43.2° can be indexed to the (002) and (110) planes of MWCNT [38,56]. The particle size was measured as 5 nm. The properties of the broad silica peak were observed at 2θ = 22° and illustrated the effect of amorphous silica [57,58].

## 3. Experimental Procedures

### 3.1. Dispersing the Nanoscale Uniformly in Water, and Workability

Untreated nanoparticles are prone to aggregate (firmly-held clusters) due to the existence of considerable Van der Walls force along with the electrostatic force within the nanoparticles [59]. Moreover, such aggregation can cause defects in the matrix and reduce the cement paste’s strengths. Ultrasonic dispersion processing is commonly used to enhance the dispensability of nanoparticles to produce a homogeneous cement paste [60]. According to previous studies, ultrasonic dispersion significantly improves the dispersing effect [61]. In this context, an ultrasonic disrupter was used in the present research to decrease the nanoparticles’ agglomeration. The total allocated time for the sonication was 15 min. Nanoparticles were added to distilled water and evenly stirred to be wetted completely. MWCNT were dispersed using a dual cavitation process, which is stirred for 10 min and then was ultra-sonicated for 5 min at room temperature (25 °C) with one-third of the mixing water. After stirring the nanoparticles in water, the ultrasonic disrupter (750 W, 20 kHz) was used to decrease the nanoparticles’ agglomeration in the water. The dispersant agent and defamer were dissolved in water. The JJ-5 type cement mixer with 140 and 285 rpm rotation rates along with the ZS-15 type vibrator (Wuxi Jiangong Test Co., Wuxi, China) were used for mixing the cement and nanoparticles’ suspension. In the dispersion process, the suspension was kept in ice water to avoid foaming and heating during the sonication process. After the completion of the mixing process, the cement paste’s workability was evaluated by performing a mini- slump test considering the limited amount of produced cement paste. The mini-slump test was performed according to the method proposed by Collins et al. [62]. In this matter, a miniature slump cone was used with a height of 57 mm and bottom and top diameters of 38 and 19 mm, respectively. The slump diameter was reduced by 37.8% at *w*/*c* = 0.3 by adding 2% SiO_2_ and 0.3% CNT to the cement paste. Therefore, the Polycarboxylate-based superplasticizer and the remaining water were added into the mixer to improve the workability [63,64].

### 3.2. Preparation of Cement Paste

After the nanomaterials were dispersed in distilled water, the OPC cement was added to the mixture. The mixing operation was performed simultaneously for 2 min at high speed (140 rpm) in the mixer. As an additional effort to effectively increase the dispersion of nanoparticles, the cement paste was stirred for an extra 2 min at the speed of 285 rpm. The prepared specimens are summarized in Table 3.

### 3.3. Specimen Preparation

The well-mixed cement paste was poured into the metal molds (50 mm × 50 mm × 50 mm) for compressive tests, and prisms with a size of 50 mm × 50 mm × 180 mm were used for flexural tests. The specimens were maintained in the laboratory, standard chamber, for 24 h at 23–25 °C and 95% humidity. The exposed surfaces were sealed using plastic sheets to avoid possible moisture loss. After this period, the molds were removed vertically to ensure no lateral disturbance could occur. In the next step, the specimens were kept in distilled water at the same temperature. The specimens’ storage time under these conditions was recorded in 7- and 28-day intervals. Table 3 also shows the percentages of the used NS and MWCNT in the specimens.

### 3.4. Testing Methods

The compressive testing was conducted at 7 and 28 days after preparing the cubic specimens. The hydraulic mechanical testing system (MTS) was used to apply the controlled compressive load. The compressive strength was performed according to the ASTM C109/C109 M-11b specifications [65]. Strengths of three samples for each mixture design were measured, and the average value is reported as the compressive strength. In addition, the flexural capacity of specimens was measured using the bend tester to apply the controlled load on the long surface of prism specimens based on the ASTM C348-20 standard [66]. The load application on specimens was performed for 50–90 s using a maximum control rate of 0.1 mm/min. Similarly, the average values of the bending tests for each mixture were documented. After performing the mechanical tests, selected crushed specimens were used for conducting the SEM test. The SEM test was performed by S-4700 type, Hitachi, Chiyoda, Tokyo, Japan, device for characterization of nanoparticles’ distribution in the matrix and distilled water. The SEM samples were acquired as remaining pieces with dimensions of 3 mm × 5 mm × 5 mm from the cube which was subjected to compression. Prior to the SEM test, a very thin layer (1 nm) was used to sputter-coat the fracture surface.

## 4. Results and Discussion

In this section, the compressive and flexural strengths of the cement paste at the ages of 7 and 28 days using different wt.% of NC and MWCNT are discussed. Moreover, analyses of element distribution in the specimens along with the size and location of NS and MWCNT in the specimens are presented using SEM images.

### 4.1. Compressive Strength Test

**Figure 3:**
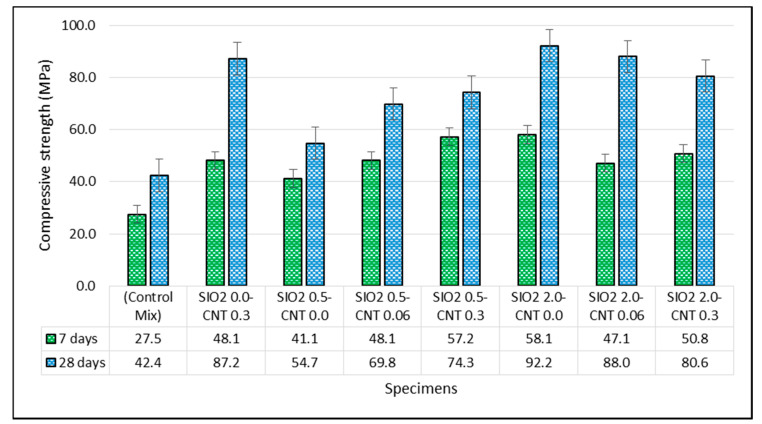
Compressive strength of cement paste specimens at the ages of 7 and 28 days.

Figure 3 displays the measured compressive strength of specimens according to the experiments at the ages of 7 and 28 days. The results show that considerable changes in compressive strength were observed by adding nanoparticles to cement paste. In 7-day specimens, the test outcomes reveal that adding MWCNT could enhance the specimens’ compressive strength, compared to the control mix. In this period, the highest increase was observed for specimens SiO_2_ 2.0–CNT 0.0 and SiO_2_ 0.5–CNT 0.3 with 111% and 108% improvements, respectively. In these specimens, MWCNT and NS had various wt.%. The equal compressive strength of SiO_2_ 0.5–CNT 0.3 and SiO_2_ 0.5–CNT 0.06 indicates that variations of MWCNT cannot necessarily affect the cement paste’s mechanical property by using 0.50 wt.% of NC.

In 28-day specimens, the results indicate that the addition of NS improved the compressive strength of the specimens. Such enhancements were more significant in specimens SiO_2_ 2.0–CNT 0.06, SiO_2_ 2.0–CNT 0.0, and SiO_2_ 0.0–CNT 0.3 compared to the other specimens. The greatest enhancement of compressive strength is reported in specimen SiO_2_ 2.0–CNT 0.0 with a 117% increase. Furthermore, it was observed that the compressive strength in specimens SiO_2_ 0.0–CNT 0.3 and SiO_2_ 2.0–CNT 0.06 are very close to each other, with 105% and 107% of improvement, respectively. Thus, adding NC in the mixture cannot ensure a distinguishable improvement in the cement paste’s mechanical behavior in the presence of MWCNT.

On the other hand, by adding NS, the compressive strength was increased slightly more in the 28-day specimens compared to the-day specimens. In 7-day specimens, the ultimate increase in compressive strength was up to 58 MPa, which is a significant improvement compared to the control specimen with a strength of 27.5 MPa. Similarly, in the 28-day specimens, compressive strength was increased by almost 50 MPa. NS particles also improve the pozzolanic activity and reduce the porosity, which results in a more homogenous, compact, and denser microstructure, thus enhancing the compressive strength [67].

Comparing the measured values of the compressive strength shows that, for the constant amount of MWCNT equal to 0.3%, in the presence of NS varying between 0% and 2.0%, the 28-day compressive strength was improved up to 87.2 MPa. In addition, it was observed that the maximum compressive strength value was achieved in the absence of MWCNT, as is reported for specimen SiO_2_ 2.0–CNT 0.0. This finding shows that the combination of NS and MWCNT cannot necessarily improve the compressive strength of concrete. In this regard, the presence of MWCNT as an additive to the cement with NS could adversely affect the compressive strength. The accelerating effect of adding NS to the cement paste was indirectly studied by controlling the changes in viscosity and rheological measurements [68]. Measurements of viscosity illustrate that incorporation of NS in the mortar and cement paste increases the water demand to maintain the same level of workability. Accordingly, Quercia et al. [69] asserted the direct relationship between water demand and workability. The high water demand of NS in the mixture can cause the negative effect on the mechanical characteristics of specimens, as shown in the results of the present study. Such a negative effect can also influence the degree of improvement with the binary use of MWCNT and NS in the cement paste.

Moreover, as reflected in this research, the use of MWCNT can lead to a partial reduction in the strength, despite increasing the MWCNT dosage. Accordingly, Sobolkina et al. [70] stated that the bridging of C-S-H phases varies based on the CNT type, which, to some extent, changes the effectiveness of incorporating CNT on mechanical characteristics of cement paste. Moreover, the presence of Van der Waals’ force hinders uniform dispersion of CNT. Without a uniform dispersion, incorporating CNT cannot lead to promising results on improving the strength of cement paste. Zhou et al. [71] addressed this issue by using graphene oxide (GO) as a dispersant in the presence of CNT. The use of GO leads to pores refinement in the cement paste. The negative effect of CNT can be addressed by modifying the CNT’s polymeric matrices in concrete. For instance, Güler et al. [72] modified the surface of CNT using polymeric surfactants such as Poly (ethylene glycol) (PEG), Polyvinyl alcohol (PVA), Dodecylamine-(DDA), and Polyvinylpyrrolidone (PVP) in an attempt to enhance concrete’s mechanical properties. The use of such polymeric surfactants avoids the requirement for additional proves of CNT’s surface, compared to other recommended different surfactants [73,74]. By implementing this modification, the strength can be improved up to 60%, even for low dosages of CNT such as 0.05 wt.%. The PVP surfactants had the most considerable influence on strength values compared to the two other stated surfactants.

### 4.2. Flexural Strength Test

**Figure 4:**
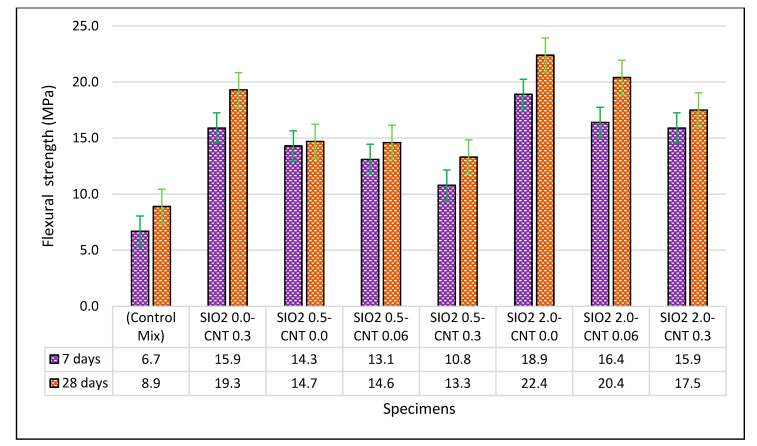
Flexural strength of cement paste specimens at the ages of 7 and 28 days.

The reported flexural strength values of the specimens in the 7- and 28-day periods are presented in Figure 4.

For the seven-day specimens, it was observed that adding NS and MWCNT improved the flexural strength, compared to the control mix. For example, 182% enhancement for specimen SiO_2_ 2.0–CNT 0.0 was observed. In this specimen, the notable increase in the flexural strength is recorded in the absence of MWCNT for a7-day period. This improvement with the highest percentages of NS can be attributed to excluding MWCNT from the cement paste. In this regard, some investigations revealed that the sliding of MWCNT from the paste’s matrix during tensile and flexural loads causes a weak bond between MWCNT and the matrix [75], which cannot improve the strength of cement paste containing NS. Furthermore, it was observed that, during the 28-day curing period, adding different wt.% of nanoparticles to the cement paste improved the flexural strength, in comparison to the control mix. In this period, the minimum of 49.4% improvement and the maximum of 151% enhancement of flexural capacity were recorded by adding nanoparticles. The most remarkable flexural strength after 28 days of improvement was measured in specimen SiO_2_ 2.0–CNT 0.0. The flexural strength of the specimens at the age of 28 days showed a comparable improvement trend with the 7-day specimens. Moreover, it was found that the flexural strength declined when adding more dosages of MWCNT for the specimens with the same 2.0 wt.% of NS, e.g., specimens SiO_2_ 2.0–CNT 0.06 and SiO_2_ 2.0–CNT 0.3.

The similar trend in reduction of flexural strength was observed for the samples with the constant 0.5 wt.% of NS containing MWCNT. For instance, by addition of MWCNT, from specimen SiO_2_ 0.5–CNT 0.0 to specimen SiO_2_ 0.5–CNT 0.3, the flexural strength was reduced by 4.7 MPa. This reduction in the flexural strength highlights the fact that, notwithstanding the high tensile strength of MWCNT, its effect on the flexural strength is only evident if MWCNT is separately incorporated. By adding MWCNT to the cement paste in the presence of NS, the flexural strength is reduced in the case of using SiO_2_ 2.0 or SiO_2_ 0.5, considering the comparable error margins of experiments. This finding displays the fact that the synergistic effect of using these nanomaterials in the cement past cannot always be positive. Aydin et al. [30] attributed the negative effect of CNT to its high water demand. They stated that the use of fly ash in addition to MWCNT and NS can address this issue. The outcome of the bending tests in the present study shows that such a binary incorporation of MWCNT and NS cannot always have the most influential effect on improving the cement paste’s flexural strength.

In addition, the flexural strength for specimen SiO_2_ 0.0–CNT 0.3 shows a considerable improvement compared to the control mix during 7- and 28-day curing periods. The enhancement shows the positive effect of MWCNT, if NS is excluded from the cement paste. In this regard, Wang et al. [32] reported that MWCNT bridges across cracks and pores in the matrix. Following the FESEM analysis, they observed that during tension loads, MWCNT was pulled out from the cement paste’s matrix. This response enhances the load-transfer during tension, which in turn increased the flexural strength, thus confirming the positive effect of MWCNT on the flexural strength. These observations show that the use of NS cannot always have promising effect on the flexural properties in the binary use with the MWCNT.

### 4.3. Scanning Electron Microscope (SEM)

**Figure 5:**
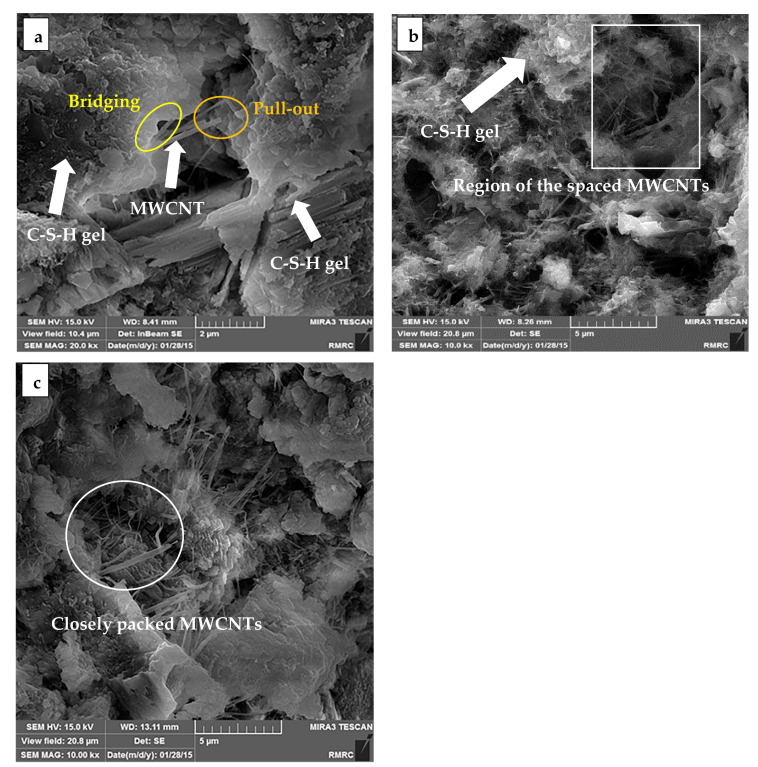
SEM images of the cement paste with w/c of 0.3: (**a**) bridging and pull out response of the MWCNT in for the SiO_2_ 0.5–CNT 0.06 specimen; (**b**) carbon nanotube’s distribution in the prepared SiO_2_ 0.5–CNT 0.06 specimen; and (**c**) Ca(OH)_2_ crystals developed from hydration in the SiO_2_ 2.0–CNT 0.3 specimen.

SEM analysis was performed to assess the MWCNT dispersion quality and interaction of these nanoparticles in the cement paste. The evaluated dispersion concentrations were 0.0 and 0.3 wt.% CNT for specimens SiO_2_ 0.5–CNT 0.06 and SiO_2_ 2.0–CNT 0.3. The typical length for MWCNT in the experiment was between 5 and 15 µm. Possible damages of MWCNT during the dispersion process can reduce its length. Figure 5 illustrates the size and dispersion of the incorporated MWCNT with different dosages in the cement paste. Considering the special characteristics of MWCNT, such as its size and high aspect ratio (length to diameter), this nanoparticle can be more efficiently distributed in very fine scales, compared to other reinforcement materials, which in turn enhances the strength of cement paste by bridging the cracks at initial stages of crack propagation in the matrix. Figure 5a shows an individual MWCNT along with its micro-crack bridging and pull out behaviors. MWCNT are more distinguishable at early ages in that the cement hydration products can be further attracted by the large surface energy of this nonmaterial over the curing time. It was observed that an attraction by the C-S-H gel forms a coat over MWCNTs and mostly obscures them from detection. The combination of C-S-H and MWCNT, which can be orientated in various directions, leads to the formation of a multiphase product that contributes to enhancing the mechanical characteristics of the cement paste. The pull out response of MWCNT facilitates the load transfer in the cement paste and improves the flexural strength. MWCNTs should be uniformly dispersed within the cement paste to further enhance the strength characteristics in the matrix. Despite the use of an ultrasonic disrupter, some agglomeration and deficiencies in the binary use of MWCNT and NS within the cement paste were observed, which adversely affects the compressive and flexural strengths of the tested specimens (Figure 5b,c).

## 5. Conclusions

In this study, silica nanoparticles and carbon nanotubes with different wt.% were added to Portland cement paste to investigate their effect on improving the cement paste’s mechanical properties. The primary outcomes of this study are summarized as follows:

- Following the results of conducted experiments in this study, the binary incorporation of MWCNT and NS cannot ensure the most influential effect on enhancing the cement paste’s compressive and flexural strengths.
- The compressive strength of the cement paste was improved by adding nanoparticles, compared to the control mix. This enhancement was slightly higher in the 28-day specimens compared to the 7-day specimens. The maximum improvement at the ages of the 7 and 28 days were 111% and 117%, respectively.
- The most considerable improvement in compressive strength was measured by using 2 wt.% of NS in the absence of MWCNT. This observation indicates that adding the NS in the presence of MWCNT cannot always result in the most favorable strength enhancement. Accordingly, the high water demand of MWCNT can adversely affect the mechanical characteristics of cement paste containing NS.
- Increasing the MWCNT dosage in the paste with a constant amount of NS led to a partial reduction in the compressive strength.
- Compared to the plain cement paste, incorporating MWCNT and NS enhanced the flexural strength of specimens for both the 7-day and 28-day curing periods. However, based on the measurements, the flexural strength increased up to 182% in the seven-day as well as 151% in the 28-day specimens if MWCNT was excluded. These comparable results for both curing periods can be attributed to the weak bond of MWCNT in the presence of NS and its sliding performance in the matrix during the flexural loading.
- The reported flexural strengths show further enhancement by using MWCNT and excluding NS in the curation intervals. In this regard, the pull out response of the MWCNT facilitates the load-transfer during tension, which improves the flexural strength.
- The reported trends of reductions in the compressive and flexural strengths using the same percentages of NS and adding different percentages of MWCNT are comparable at similar error margins. These strength reductions indicate that, despite the high tensile strength of MWCNT, its effect on the flexural strength is only evident if MWCNT is separately incorporated.
- The SEM analysis showed bridging and pull out responses of MWCNT, which contributed to the enhancement of the cement paste’s strength.
- It is recommended to further investigate the synergistic effect of nanoparticles in addition to other materials with different combinations in an effort to highlight not only the most promising outcomes, but also adverse influence of the binary or ternary use of nanoparticles.

## Figures and Tables

**Table 1:** Chemical compositions of the Portland cement type II (wt.%).

Material	Al_2_O_3_	SiO_2_	MgO	CaO	Fe_2_O_3_	K_2_O	Na_2_O	SO_3_	LOI
Portland Cement	4.8	21.3	2.4	64.0	3.9	0.7	0.4	2.1	1.1

**Table 2:** Material properties of multi-walled carbon nanotubes (MWCNT) and nanoparticles SiO_2_ (NS).

Product Name	Appearance (Color)	Diameter (nm)	Specific Surface Area (m^2^/g)	Purity (%)	Length (µm)
Multi-walled carbon Nanotubes (MWCNT)	Black	20–40	80–140	>98	5–15
Nanoparticles SiO_2_ (NS)	White Powder	7–40	300	99.8	---

**Table 3:** Prepared specimens of the mixture design.

Specimen ID	NS (wt.%)	CNT (wt.%)	Water (g)	OPC (g)	NS (g)	CNT (g)
1 (Control Mix)	0.00	0.00	380	172	0.00	0.00
2 SiO_2_ 0.0–CNT 0.3	0.00	0.30	380	172	0.00	0.51
3 SiO_2_ 0.5–CNT 0.0	0.50	0.00	380	172	0.86	0.00
4 SiO_2_ 0.5–CNT 0.06	0.50	0.06	380	172	0.86	0.10
5 SiO_2_ 0.5–CNT 0.3	0.50	0.30	380	172	0.86	0.51
6 SiO_2_ 2.0–CNT 0.0	2.00	0.00	380	172	3.44	0.00
7 SiO_2_ 2.0–CNT 0.06	2.00	0.06	380	172	3.44	0.10
8 SiO_2_ 2.0–CNT 0.3	2.00	0.30	380	172	3.44	0.51

## Data Availability

The data presented in this study are available on request from the corresponding author.
